# Lymphangioma Presenting as Juxta-Articular Swelling In Children: A Case Series

**Published:** 2013-07-14

**Authors:** Deepak Jain, Harpal Singh Selhi, Mohd Yamin

**Affiliations:** Dayanand Medical College and Hospital, Ludhiana, India

Lymphangiomas are rare congenital malformations arising due to the failure of development of communications between lymphatic channels with subsequent sequestration and dilatation of lymphatic tissue. Majority of these lesions are seen in the neck and facial region while other sites such as axilla, mediastinum, retroperitoneum, mesenteric and groin have also been reported [1]. The occurrence of lymphangiomas in upper or lower limbs has been published in a few case reports [2,3]. We report our experience of treating lymphangiomas presenting as isolated juxta-articular swellings in paediatric population. These lesions can be confused with intra-articular swellings / effusions and need to be differentiated from them. The clinical features, diagnostic workup and operative findings of the cases in our series are given in Table 1. All the patients underwent surgical excision for these swellings under regional or general anaesthesia. The average time of follow up after surgery was 10 months (range 6 to 14 months). There was no post operative infection, recurrence or any other complication in any patient.

**Figure:**
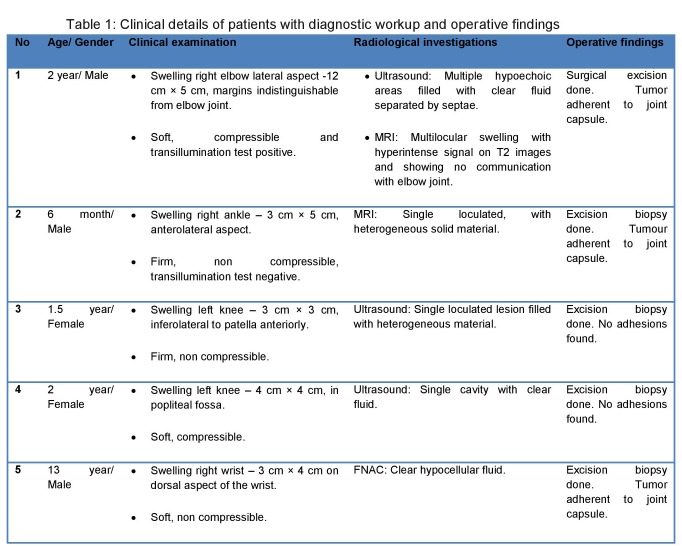
Table 1

The clinical presentation of the cases in our series is different from those reported in literature. All the cases presented with isolated swelling of the affected joints noted either at birth or within subsequent three months. On examination, they were eccentrically located in proximity of the joints which helped differentiate them from joint effusion. They were soft on palpation except in cases 2 and 3 which were firm and in variance with the typical presentation of lymphangiomas. Transillumination which is characteristic of lymphangioma was noted in only case 1 as the other lesions were small or had solid contents. Ultrasonography was done to find out the relation of the swellings with the joints. MRI was done in cases 1 and 2 to ascertain whether the lesions communicated with the joints and their relationship with neurovascular structures.

In case 1, it was possible to make a pre operative diagnosis of lymphangioma based on clinical findings and diagnostic workup (Fig. 1). In the rest of the cases, excision biopsy was done and the diagnosis was established after histopathological examination. In three cases (Cases 1, 2 and 6) lymphangioma was adherent to the joint capsule in some areas and partial excision of joint capsule and synovium was required to ensure complete excision of the tumour. The typical finding of thin tumour sheaths filled with clear fluid was not present in cases 2 and 3 which were solid tumours filled with heterogeneous cellular material (Fig. 2). This is possible if spontaneous haemorrhage occurs in the cystic cavity with subsequent fibrosis. In our series, all the cases were macrocystic type of lymphangiomas except case 1 which was of mixed type.

**Figure:**
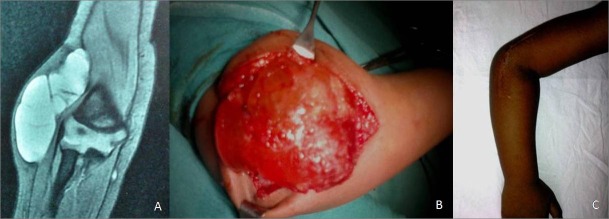
Figure 1 (A, B, C): A- MRI of elbow showing multilocular swelling with hyperintense clear signal, B-. Operative photograph showing typical findings of multilocular swelling with thin walls, C- photograph showing follow up at one year.

**Figure:**
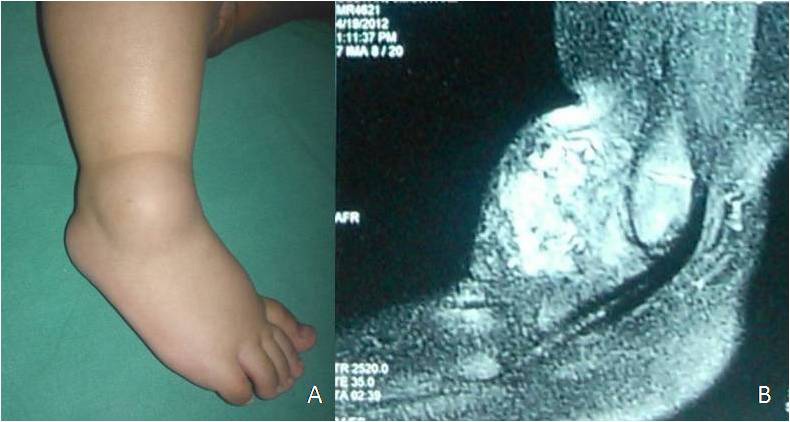
Figure 2:(Case 2) Clinical photograph of ankle swelling (A) and MRI of ankle showing solid tumour with heterogeneous signal (B).

Surgical excision is the mainstay of treatment of lymphangiomas. Sclerotherapy is also used to treat such lesions and agents like bleomycin, doxycycline and OK 432 have been used particularly in large lesions and those located in inaccessible areas.[2,4]. Ozeki et al have advocated the use of propranolol (a beta blocker) as an adjunct in treating intractable lymphatic malformations. [5] Vaccum assisted closure has also been used to assist the closure of wounds after surgical excision.[6] However, in our series all the lesions were isolated with well defined margins and showed excellent results with surgical excision. 

## Footnotes

**Source of Support:** Nil

**Conflict of Interest:** None declared

